# Preparation and Characterization of Epoxy Resin Cross-Linked with High Wood Pyrolysis Bio-Oil Substitution by Acetone Pretreatment

**DOI:** 10.3390/polym9030106

**Published:** 2017-03-15

**Authors:** Yi Liu, Brian K. Via, Yuanfeng Pan, Qingzheng Cheng, Hongwu Guo, Maria L. Auad, Steven Taylor

**Affiliations:** 1 MOE Key Laboratory of Wooden Material Science and Application, Beijing Forestry University, Beijing 100083, China; 2 Beijing Key Laboratory of Wood Science and Engineering, Beijing Forestry University, Beijing 100083, China; 3 MOE Engineering Research Center of Forestry Biomass Materials and Bioenergy, Beijing Forestry University, Beijing 100083, China; 4 Forest Products Development Center, School of Forestry and Wildlife Sciences, Auburn University, Auburn, AL 36849, USA; ypan@uzz.edu.cn (Y.P.); qzc0007@auburn.edu (Q.C.); 5 School of Life Sciences, Zaozhuang University, Zaozhuang 277160, China; 6 Department of Chemical Engineering, Auburn University, Auburn, AL 36849, USA; aza0082@auburn.edu; 7 Department of Biosystems Engineering, Auburn University, Auburn, AL 36849, USA; taylost@auburn.edu

**Keywords:** wood pyrolysis bio-oil, epoxy, acetone pretreatment, cross-linking, substitution

## Abstract

The use of cost effective solvents may be necessary to store wood pyrolysis bio-oil in order to stabilize and control its viscosity, but this part of the production system has not been explored. Conversely, any rise in viscosity during storage, that would occur without a solvent, will add variance to the production system and render it cost ineffective. The purpose of this study was to modify bio-oil with a common solvent and then react the bio-oil with an epoxy for bonding of wood without any loss in properties. The acetone pretreatment of the bio-oil/epoxy mixture was found to improve the cross-linking potential and substitution rate based on its mechanical, chemical, and thermal properties. Specifically, the bio-oil was blended with epoxy resin at weight ratios ranging from 2:1 to 1:5 and were then cured. A higher bio-oil substitution rate was found to lower the shear bond strength of the bio-oil/epoxy resins. However, when an acetone pretreatment was used, it was possible to replace the bio-oil by as much as 50% while satisfying usage requirements. Extraction of the bio-oil/epoxy mixture with four different solvents demonstrated an improvement in cross-linking after acetone pretreatment. ATR-FTIR analysis confirmed that the polymer achieved a higher cross-linked structure. DSC and TGA curves showed improved thermal stability with the addition of the acetone pretreatment. UV-Vis characterization showed that some functional groups of the bio-oil to epoxy system were unreacted. Finally, when the resin mixture was utilized to bond wood, the acetone pretreatment coupled with precise tuning of the bio-oil:epoxy ratio was an effective method to control cross-linking while ensuring acceptable bond strength.

## 1. Introduction

The clean and efficient utilization of renewable biomass has already attracted unprecedented concern from the general society. Fabrication of wood bio-oil, through fast pyrolysis, is an alternative method to produce petroleum-based feedstock and also a viable and effective alternative for building bio-based materials [1,2,3]. Efforts have been made to study the wood pyrolysis process and its effects on bio-oil quality, product regulation and refining, and composite manufacture [4,5,6,7]. Wood pyrolysis bio-oil is an attractive feedstock given the large volume of active phenolic hydroxyl, carboxylic acid, and carbonyl functional groups [8]. The use of bio-oil, as a replacement for phenols in bio-derived or bio-substituted resins, is a desirable goal in the displacement of petroleum based feed stocks. Further, it can provide a pollution-free environment and biodegradation during the waste portion of the lifecycle. As a result, wood bio-oil has the potential to become a significant component in future novel resins and composites.

Phenol formaldehyde (PF), melamine formaldehyde, and polymeric diphenylmethane diisocyanate (pMDI) resins are the most common resins used in the wood composite industry. As such, substitution of wood bio-oil and/or lignin into these resins has been well studied. Studies of bio-oil/PF resin systems found that bio-oil provided many different oxygenated organic compounds, such as phenolic hydroxyl groups, which are important during curing and adhesion [5,9,10,11]. The manufacture of bio-based PF resins revealed that bio-oil could participate in the phenol-formaldehyde reaction [5]. Amen-Chen et al. investigated the curing process of PF resins with a 25 wt % of phenol replaced with bio-oil [12]. It was found that the curing mechanism of the bio-oil/PF resin was comparable to that of pure PF. On the other hand, an increase in bio-oil content slowed the curing kinetics and reduced the thermal resistance of the resin [12]. This has provided to be a significant barrier to industrial usage where hot-press time is a critical factor in the overall cost structure. Substitutions of up to 50% of the phenolic component with bio-oil in resole-type resins have been achieved in oriented strand board (OSB) and plywood production [13]. It was also found that higher phenol substitution rate is likely if the fraction is enriched in reactive phenolics. Zheng successfully produced a phenolic resin with a 30 wt % of bio-oil, and its mechanical performance was reached as required by the Chinese National Standard (GB/T 14732-2006) [14]. Likewise, in another study, it was reported that most of the chemicals in bio-oil were able to react with pMDI, although a substantial part is not highly suitable as cross-linking agents and adhesion promotion. Gagnon et al. studied the feasibility of using bio-oil as part of a pMDI resin system for particleboard [15,16]. They suggested that bio-oil could replace as much as 40 wt % of pMDI while maintaining acceptable interior grade particleboard properties. Mao et al. mixed bio-oil with pMDI and added acetone into the bio-oil/pMDI resin prior to making flakeboard [17]. Their results showed that incorporation of acetone into pMDI reduced the viscosity by 67% and this improved the uniformity of the bond lines between the flakes resulting in a higher internal bond (IB). However, the IB was significantly reduced at substitution rates greater than 25%.

Epoxy resin is an alternative candidate for wood bonding given it has a strong dry strength, low addition ratio, and excellent environmental tolerance. However, the issue of adhesion to the press platen, higher costs, and increased brittleness limit its application in the wood industry [18,19]. Furthermore, the linkage between the wood substrate and epoxy can be considerably weakened after exposure to water. This is due to the forces at the bondline becoming high during swelling and these forces exceed the interfacial strength [20]. However, the addition of bio-oil to wood helps to resist moisture and consequent swelling [21]. It may thus be possible for bio-oil to interact with the wood substrate during pressing resulting in better resistance to moisture exposure at the bondline while simultaneously cross-linking with the epoxy while curing [3,22,23].

Recently, some researchers have attempted to substitute alcohol-liquefied wood into the epoxy resin system for particleboard [24,25,26]. They found the alcoholic –OH groups of the liquefied wood reacted with epichlorohydrin under alkaline conditions. Their results demonstrated that wood-derived molecules were chemically incorporated into the resin to form a cross-linked copolymer network structure. The liquefied wood/epoxy resin presented a shear bonding strength similar to commercial epoxy resin [27,28]. Wu and Lee mixed liquefied wood with epoxy resin at various weight ratios and studied its curing behavior and adhesion properties [26]. Their results showed that the blended epoxy resin had a good dry bond strength for wood when cured at room temperature.

The degree of cross-linking within the resin is an important factor that influences the bonding ability of the modified resin. This paper discusses the usability of a common epoxy resin that has been blended with commercially available wood pyrolysis bio-oil. A range of bio-oil:epoxy ratios were studied and the effect of an acetone solvent pretreatment on the bio-oil/epoxy resin performance was investigated. This work provided a simple and effective method to develop a novel epoxy resin cross-linked with wood pyrolysis bio-oil for wood and other polymer composite industries.

## 2. Materials and Methods

### 2.1. Materials

Wood pyrolysis bio-oil was produced at Red Arrow International (Manitowoc, WI, USA) which operates a circulating fluidized bed at 1500 to 1700 kg/h (40 to 45 tons/day) [29,30]. The feedstock was comprised of the common American hardwoods used for the manufacture of liquid smoke [31]. Wood pyrolysis bio-oil is a multi-component mixture of different sized molecules derived from depolymerization and fragmentation of cellulose, hemicellulose, and lignin. Over 99% of the bio-oil is composed of water, acids, alcohols, aldehydes, esters, ketones, phenols, guaiacols, syringols, furans, lignin-derived phenols, and extractible terpene with multifunctional groups [17,32]. The water was removed prior to shipping from the manufacturer. Upon acquisition, the crude bio-oil was stabilized and homogenized with the addition of 100% *V*/*V* reagent-grade methanol. Methanol is a common solvent for phenolic compounds. It provides a means to reduce polymerization of the bio-oil feedstock and prevent degradation of volatile components [1,21]. The diluted bio-oil was vacuum-filtered through #1 Whatman paper to separate the particulate solids, and then vacuum rotary distilled at 70 °C to remove the methanol. After that, the bio-oil was stored in a refrigerator at 4 °C before use.

Epon 828 (Catalog No. NC9610653, Manufacturer: E. V. Roberts and Associates Inc. 174-1 gallon, Fisher Scientific, Pittsburgh, PA, USA) is a type of low molecular weight (350 to 400) bisphenol A/epichlorohydrin derived liquid epoxy resin. The resin was stored at 25 °C in a sealed container under a relative humidity of 60% to 65%. A commercial Hexion 13BO33 Liquid phenol-formaldehyde (PF) resin [33] was used as a comparison. Diethylenetriamine (99%, DETA, CAS No. 111-40-0) curing agent was purchased from VWR Chemical Co. (produced by Alfa Aesar Company, Tewksbury, MA, USA).

### 2.2. Acetone Pretreatment

Epon 828 (Bisphenol-A precursor) was blended with bio-oil at weight ratios of 2:1, 1:1, 1:2, 1:3, 1:4, and 1:5 (*m*_bio-oil_:*m*_Epon 828_), respectively. The acetone pretreatment of the mixtures was carried out in a 250 mL glass flask equipped with a magnetic rotor and reflux condenser device. The flask was put into a glass water tank, and then the tank was placed on a heated magnetic stirrer. The bio-oil/epoxy mixture (50 g) and acetone solvent (50 g) were put into the flask reactor separately. After 5 min of stirring, during which the acetone assisted in dissolving the mixture, the flask reactor was preheated to 50 °C for 30 min and then the temperature was raised to 80 to 85 °C at a heating rate of 1 °C/min. Complete reaction was achieved within 2 h at which time a rotary vacuum was used at 60 °C to remove the solvent while being stirred constantly. These homogeneous mixtures were then cooled down to 23 °C and stored in a cool, dry place without light.

### 2.3. Preparation of Bio-Oil/Epoxy Resins

Diethylenetriamine (DETA) was added as a catalyst into the bio-oil/epoxy mixtures and pure Epon 828 at 10% by weight of Epon 828 was also added. Tetrahydrofuran, which can quickly evaporate, was used as a solvent to minimize air bubbles within the resin for uniform mixing and curing. Samples for the following tests were performed by pouring 25 g of pure Epon 828 and bio-oil/epoxy mixture at each ratio into aluminum dishes (Ø 56 mm), respectively. Curing temperatures and ramp schedules of 60 min at 50 °C, 30 min at 80 °C, and 120 min at 100 °C respectively were implemented. After curing, the samples remained in the oven to cool down slowly to 23 °C in order to prevent bubbling.

### 2.4. Properties of Bonding Strength

Loblolly pine wood (*Pinus taeda* L.) lamellas with dimensions of 80 mm × 20 mm × 5 mm were used as substrates for the preparation of two-layered specimens, which were bonded in accordance with the standard of EN 205: 2003 by hot pressing [33,34]. Prior to bonding, all of the wood lamellas were sanded with 200 mesh sandpaper to ensure a smooth and flat surface. The pure Epon 828, pretreated and untreated bio-oil/epoxy mixtures (10% DETA added), and PF were applied to one surface of one wood lamella with a consumption rate of 140 g/m^2^ using a glass roller and then the two lamellas were placed together equably. The length of the overlap portion of the joint was 10 mm. The bonded samples were completely wrapped in aluminum foil and hot-pressed at 300 F (148.9 °C) for 15 min with a pressure of 130 Psi (0.896 MPa). The specimens were stored at 105 °C for 4 h, and then cooled to 23 °C. The tensile shear resin strength was evaluated using a universal strength testing machine (Zwick Roell testXpert II, Einsingen, Germany) with a loading speed of 1 mm/min at ambient conditions (ASTM D790-10). Six replicates per ratio condition were tested. Additional replicates were added if the failure was less than 75% in the resin composite interface.

### 2.5. ATR-FTIR Analysis

Attenuated Total Reflectance-Fourier Transform Infrared Spectrometry (ATR-FTIR) was performed for characterization of functional group consumption of the bio-oil, Epon 828, and cured bio-oil/epoxy resins. All samples were oven-dried at 105 °C for 12 h and stored in a desiccator to cool down to avoid samples from absorbing moisture from the atmosphere. A small amount of the sample was applied directly onto the diamond crystal of the ATR-FTIR spectrometer (PerkinElmer Spectrum 400, Waltham, MA, USA) and the spectra was collected in transmittance mode by 32 scans in the range of 4000 to 650 cm^−1^ at a resolution of 4 cm^−1^.

### 2.6. Properties of Soluble Resistance

As the volume of cross-linking increases, the weight loss of the polymer upon solvent exposure should decrease and is thus a sensitive measure to the optimum ratio of bio-oil to Epon 828. To investigate material cross-linking, the cured pretreated and untreated bio-oil/epoxy resins were extracted by acetone, 1,4 dioxane, boiling water, and cold water, respectively. The cured polymers were milled to 20 mesh and heated in an oven at 105 °C overnight to remove the moisture. About 2 ± 0.25 g of ground samples were poured into a cellulose thimble which was then placed into a soxhlet extractor. The soxhlet was attached to a flask with 150 mL of organic solvent (acetone or 1,4 dioxane). Afterwards, the system was placed on a heater and refluxed under heat for 6 h (4 cycles/h). Finally, the obtained samples had the adhering solvent evaporated off in a fume hood and then dried in an oven at 105 °C until a constant weight was reached.

The water extractions were performed by immersing specimens in flasks filled with boiling water or cold distilled water (23 °C) for 3 h (boiling water) or 48 h (cold water) while being slowly stirred with a magnetic bar. A glass crucible and 2 ± 0.25 g per treatment were first conditioned at 105 °C for 12 h and weighted. After extraction, the residues were placed into the glass crucible, filtered by a vacuum pump, and then dried and weighed again.

The weight loss (*WL*, %) of the samples was calculated following Equation (1). The *WL* of the different blends was determined as the average of two separate replicates.

*WL* (%) = [(*m*_o_ − *m*_d_)/(*m*_o_ − *m*_r_)] × 100 (1)

where *m*_o_ is the weight of the cellulose tube (for solvent extractions) or glass crucible (for water extractions) plus the sample before extraction (g), *m*_d_ is the weight of the cellulose tube or glass crucible plus the sample after extraction (g) and *m*_r_ is the weight of the cellulose tube or glass crucible (g).

### 2.7. DSC, TGA and UV-Vis Analysis

The glass transition temperature (*T*_g_) and degradation characteristics of the cured pretreated and untreated bio-oil/epoxy resins were established with a Differential Scanning Calorimetry (TA Instruments Q2000 DSC, TA Instruments, New Castle, DE, USA). For each scan, 4.5 ± 0.5 mg of dried powder were added into an aluminum pan. The temperature schedule for the DSC was programmed from ambient temperature to −20 °C and then raised from −20 to 120 °C. A 50 mL/min nitrogen flow was used at a heating rate of 10 °C/min. For polymers, glass transition temperature (*T*_g_) is defined as the temperature at which the mechanical properties of a polymer radically changed from the glass state into high elastic state due to the internal movement of the polymer chains. The value of *T*_g_ is at the onset of the transition. As a result, the value of the glass transition temperature is directly related to the polymer mechanical properties (strength, hardness, brittleness, and elongation).

A thermal gravimetric analyzer (TA Instruments TGA Q500, New Castle, DE, USA) was used to determine thermal degradation properties of the bio-oil/epoxy resins after polymerization. A weight of 8.5 ± 1.5 mg of cured materials was placed on the Pt basket in the furnace and heated from room temperature to 850 °C at a rate 10 °C/min within a nitrogen atmosphere (flow rate of 40 mL/min). Curves of weight loss and derivative weight loss (DTG) were plotted and analyzed.

The UV-Vis spectra of extracts of pretreated resins were recorded at room temperature on a GENESYS 10S UV-Vis spectrophotometer (Thermo Scientific, Hudson, NH, USA). This study was conducted in the wavelength range from 200 to 600 nm and scans were run at a resolution of 3 nm.

## 3. Results and Discussion

### 3.1. Performance of Wood Bonded Shear Strength

**Figure 1:**
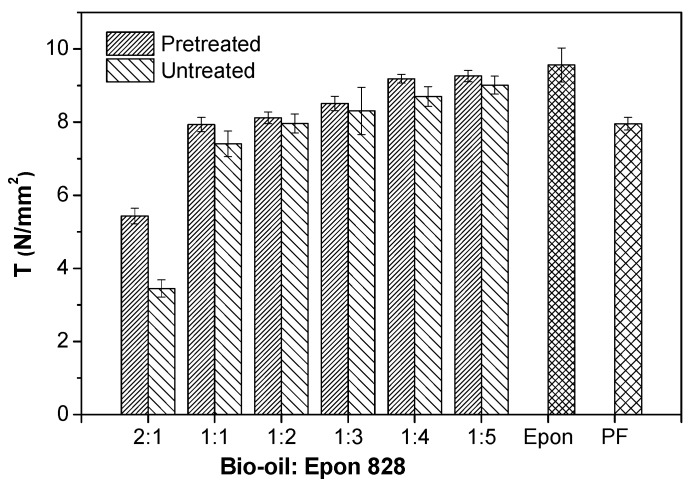
The shear strength of samples bonded with pretreated and untreated bio-oil/epoxy resins, Epon 828 and phenol formaldehyde (PF).

**Figure 2:**
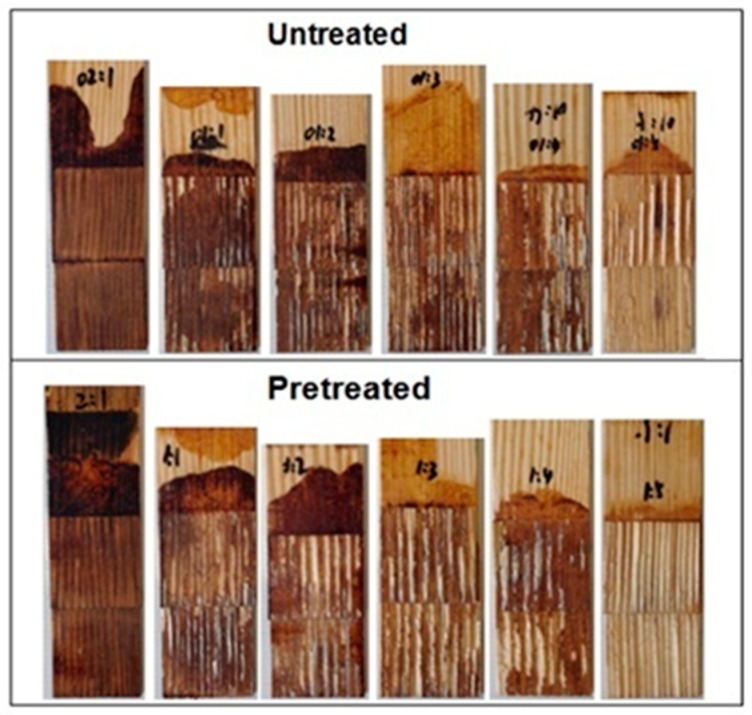
The samples bonded with untreated and pretreated bio-oil/epoxy resins.

The tensile shear strength of the tested samples bonded by the pretreated and untreated bio-oil/epoxy resins, Epon 828 control, and commercial PF control is shown in Figure 1. And Figure 2 shows tested samples bonded with untreated and pretreated bio-oil/epoxy resins. The shear strength of the bio-modified epoxy resin was heavily influenced by the ratio of bio-oil to epoxy resin. The strength decreased with the addition of bio-oil to epoxy. However, the rate of loss with bio-oil substitution was minimal and this suggests large gains in substitution may be possible. The loss in strength with substitution was an indication that increased bio-oil concentration moved away from the optimal stoichiometry ratio (1:5) for this specific system. Auad et al. supported that the optimum cross-linking density will occur when the proper molar ratio of epoxy to hydroxyl groups are blended prior to curing [35]. In this study, the decreased cross-linked density with higher amounts of bio-oil was evident based on the decreased lap joint shear strength after bonding to the pine wood substrate (Figure 1 and Figure 2).

The samples bonded with the bio-oil/epoxy resins generally exhibited higher shear strength values when the bio-oil/epoxy mixture was first pretreated with acetone. The gain in strength appears small due to the scale of the graph, but was consistently improved after pretreatment for all ratios and was on average, 9.0% higher in shear strength after pretreatment. Such a finding was novel given the purpose of the solvent was to stop viscosity rise during storage while not adversely affecting the wood composite strength. Again, the shear strength of the wood bonded joint gradually decreased as the bio-oil to epoxy ratio changed from 1:5 to 2:1 (Figure 1). On the other hand, a ratio of 1:1 (50% substitution) yielded shear strengths competitive to the commercial PF resin and exceeded the usage requirements. This suggests that the epoxy resin coupled with a bio-oil cross-linker could be competitive to PF under dry bonding conditions. However, above a 50% substitution rate, the shear strength dropped considerably and was indicative of a decline in crosslink density.

From an industrial perspective, perhaps equally as interesting was the gradual reduction in shear strength properties with an increase in bio-oil concentration. The resiliency of the system was much different than a phenol/epoxy based system in which small deviations from the optimal molar ratio resulted in a quick decrease in polymer strength [35]. It is hypothesized that when bonding wood, the additional bio-oil simultaneously cross-links to the epoxy while providing some cross-linkage to the wood substrate. According to Ugovšek and Sernek, when wood was liquefied into bio-oil and hot pressed between beech wood veneers, some natural bonding was present [34]. This tolerance to bio-oil variance is important because it suggests that industrial processes could adjust to process changes or some level of processing error.

As the replacement rate approached 50%, there was increased benefit of the pretreatment on shear strength (Figure 1). These effects were not as apparent when the bio-oil replacement rates were lower (ratios of 1:2, 1:3, 1:4, and 1:5) although a significant benefit was still observed. This finding was important because a manufacturer is more likely to operate close to the 50% substitution rate if that is the cheapest recipe at an acceptable strength.

Aside from optimal cross-linking, other factors may have contributed to the good bonding performance of the wood substrate. First, the liquid Epon 828 exhibited good fluidity and solubility with the low viscosity (110 to 150 P) necessary for adequate dispersion. This behavior was preserved for lower bio-oil loadings where dispersion into the epoxy resin did not impact viscosity too much. However, when the bio-oil content was too high (>50%), the mixture of bio-oil and Epon 828 became increasingly viscous and uniform dispersion became difficult resulting in decreased joint strength. It has been shown that the higher molecular group size of the bio-oil assists with polymerization resulting in faster curing times but heterogeneous mixtures [36]. Simple mechanical agitation was tried to homogenize the mixture but the bio-oil components were still hard to disperse and this complicated our ability to uniformly spread the mixture onto the wood surface. Second, it is theorized that the larger molecular group size of the viscous bio-oil makes it harder to diffuse the resin mixture into the substrate surface. This should result in lower bonding and consequently lower shear strength.

**Figure 3:**
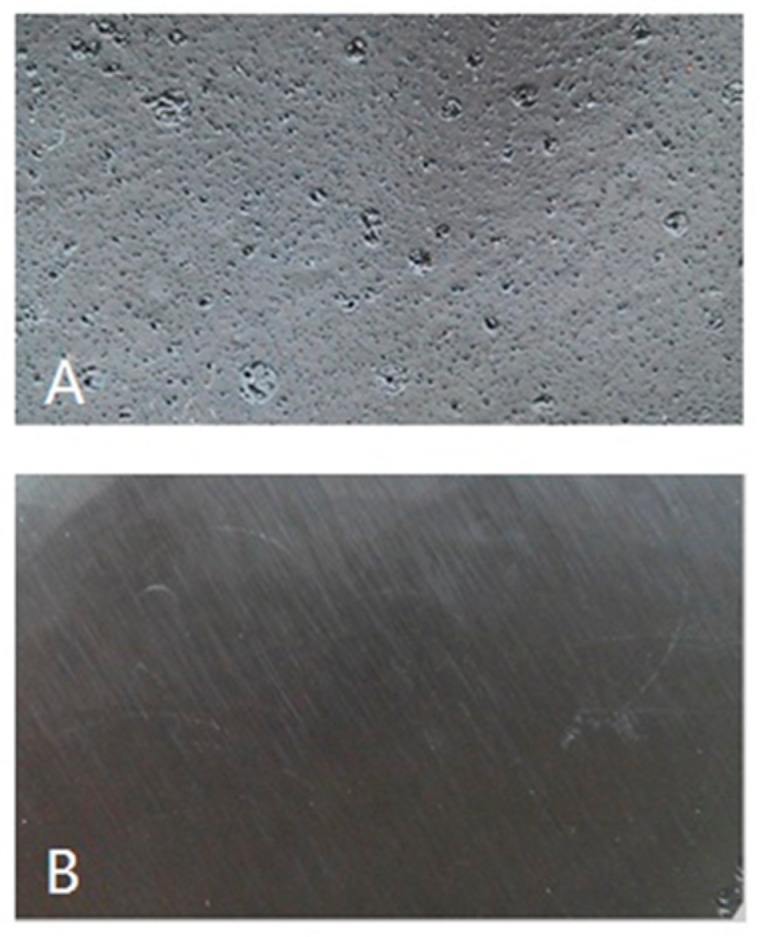
Untreated (**A**) and pretreated (**B**) cured bio-oil/epoxy resins.

Figure 3 shows the bio/oil epoxy polymer cured films. According to Figure 3A, there were also small blemishes apparent on the surface of the bio-oil/epoxy polymer. The smooth surface (Figure 3B) suggests that acetone was acting as a thinner and also a solvent for a component of the bio-oil. Acetone was successful in dissolving smaller molecular fragments that appeared to concentrate randomly across the surface for the untreated bio-oil/epoxy polymer. Further observation revealed adjacent void space, or “bubbles”, along the edge of these blemishes. These defects probably acted as a stress concentrator for the wood bonded samples as evidenced by the increased variance for the untreated group (Figure 1).

There are several possibilities for the improved homogeneity of the acetone treated bio-oil/epoxy mixture. First, it is thought that the acetone may increase the pliability of the longer molecular chains which could improve the overall contact between bio-oil active points and epoxy polymers resulting in a three-dimensional interpenetrating macromolecular network. Likewise, acetone may have assisted in the stabilization and homogeneity of the resin binder resulting in better resin diffusion into the substrate [11]. Gao and Li pointed out that the mechanical properties of wood could be reduced when exposed to acetone because acetone could decrease cellulose crystallinity and the combination interface between fibers [37]. Furthermore, it is also supposed that an acetone solvent will dissolve and reduce the molecular group size [17] resulting in higher reactivity, lower viscosity, better resin strength, and uniform colloid structure as evidenced in Figure 1 and Figure 3.

It was concluded that when the replacement rate of bio-oil was high, the pretreatment of the bio-oil/epoxy resin before synthetic reaction was necessary. A visual observation of the curing process found that pretreatment with acetone resulted in a more even curing reaction and this is thought to promote better cross-linking and improve the bonding strength.

### 3.2. ATR-FTIR Characterization

In the ATR-FTIR spectrum, the presence of bands at 3377 cm^−1^ stands for –OH stretching, 2935 cm^−1^ for –C–H stretching, 1707 cm^−1^ for C=O stretching, 1609 cm^−1^ for –OH bending, 1230 cm^−1^ for C–O stretching, aromatic C–H in plane deformation and symmetrical C–O stretching at 1029 cm^−1^, and 771 to 914 cm^−1^ for epoxide ring (–CH(O)CH–) stretching vibration. A broad absorption band at 3354 cm^−1^ was assigned to the aromatic and aliphatic –OH groups in bio-oil and are considered the primary active functional groups [38,39,40].

**Figure 4:**
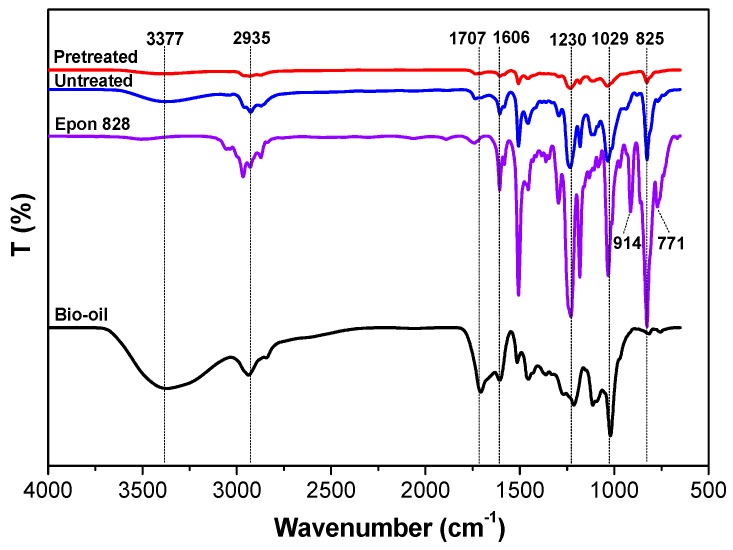
ATR-FTIR curves of bio-oil, Epon 828, untreated and pretreated cured bio-oil/epoxy resins at a 1:1 synthetic ratio.

As shown in Figure 4, after curing, the specimens of bio-oil/epoxy resin showed a significant reduction in 3377 cm^−1^ for –OH absorbance, 2935 cm^−1^ for –C–H stretching and 1029 cm^−1^ for aromatic C–H in the plane deformation absorbance [41]. The characteristic stretching vibration of the epoxide ring (–CH(O)CH–) at 914 cm^−1^ and 771 cm^−1^ showed substantial reduction after curing [42]. This confirmed the consumption of –OH groups upon reaction with Epon 828 to generate a chemical bond as a bio-based epoxy. In addition, a small peak near 1707 cm^−1^ was also noted within the bio-oil which disappeared after blending and cure. This was attributed to the carbonyl groups that were involved in the cross-linking reactions [43,44] and is represented by the following two step reaction (Equations (2) and (3)) in which this cycle was repeated until a high degree of cross-linking occurred.

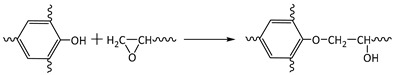
(2)

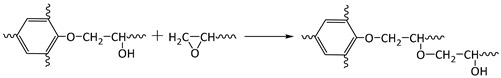
(3)

Pretreatment with acetone was found to improve –OH and –CH(O)CH– groups consumption and was indicative that acetone pretreatment to bio-oil/epoxy mixture promoted better cross-linking in the polymer. It is believed that the acetone significantly reduces the molecular group size given the drastic reduction in viscosity as seen in other studies [1]. This should open up molecular sites for reaction and improved consumption and consequent cross-linking. This may also explain the homogenous surface witnessed earlier (Figure 3B).

### 3.3. Solvent Resistance

**Figure 5:**
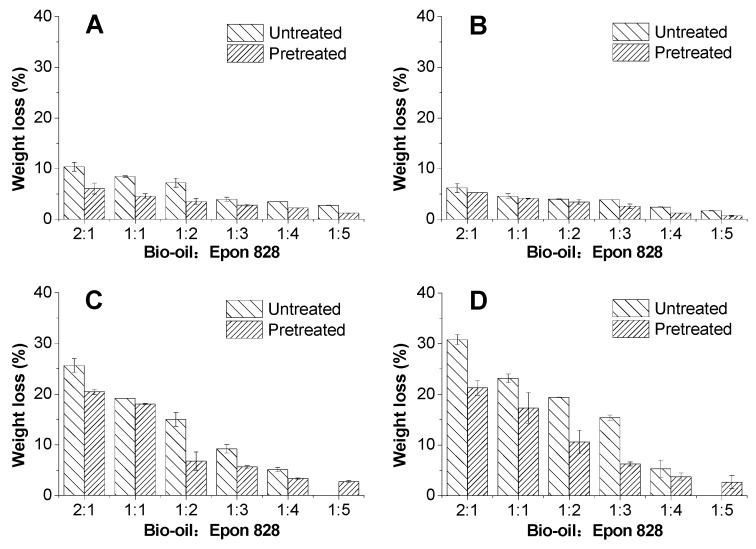
Weight loss (*WL*) of cured bio-oil/epoxy resins extracted by boiling water (**A**); cold water (**B**); acetone (**C**) and 1,4 dioxane (**D**).

The study evaluates the polymer solubility, which in turn provides an indication of the cross-linking density in cured resins. Furthermore, the solvent resistance can be used to evaluate the environmental tolerance of the cured resins and can offer some valuable data for its industrial application. The *WL* after boiling water, cold water, acetone, and 1,4 dioxane extraction tests were tested and calculated (Figure 5).

The *WL* of the bio-oil-substituted into epoxy resins decreased as the content of Epon 828 increased (Figure 5). It is noteworthy that, when the mass fraction of Epon 828 was more than 50%, the *WL* of the modified resins in the four solvents was low. But after 50% substitution with bio-oil, the *WL* increased considerably. A bio-oil:epoxy ratio of 1:5 yielded the best resistance to solvents due to better cross-linking which agreed with shear strength results (Figure 1). The increase in *WL* with bio-oil substitution may be attributed to various factors [45]. First, the cross-linking degree between the epoxy resin molecule ties up sites and resists breakdown of the molecule upon solvent exposure. Second, the availability of bio-oil –OH groups to epoxy is important in maximum cross-linking and as one moves away from the optimum molar ratio their cross-linking density will decrease. Third, there are increased concentrations of hydrophilic chemicals in the un-reacted bio-oil, such as carboxylic acids and alcohols, which tend to attract to the acetone solvent [16].

As demonstrated in Figure 5, the additional pretreatment in acetone greatly improved the soluble resistance at most ratios. This benefit was particularly noticed with higher bio-oil substitution rates. For example, when the ratio was 2:1, compared with the untreated group, the *WL* of pretreatment group decreased by 42%, 15%, 20%, and 31% in boiling water, cold water, acetone and 1,4 dioxane extraction, respectively. This means that after pretreatment of bio-oil/epoxy mixture with acetone, the bio-oil/epoxy resins became more durable and achieved better cross-linking. ATR-FTIR analysis attributed the cross-linking to be attributable to the reaction between the epoxide and hydroxyl (–OH) of bio-oil (Figure 4). When the bio-oil content is abundant, the decrease in shear strength and solvent resistance quality was offset by the promoting effect of acetone. Furthermore, the *WL* of the samples in 1,4 dioxane extraction was only 17% to 21% when the bio-oil replacement rate in the bio-oil/epoxy resins were 50% and 66.7%. This was an indication that with increased purification and refining through acetone addition, greater substitution ratios were realizable.

### 3.4. DSC Analysis

**Figure 6:**
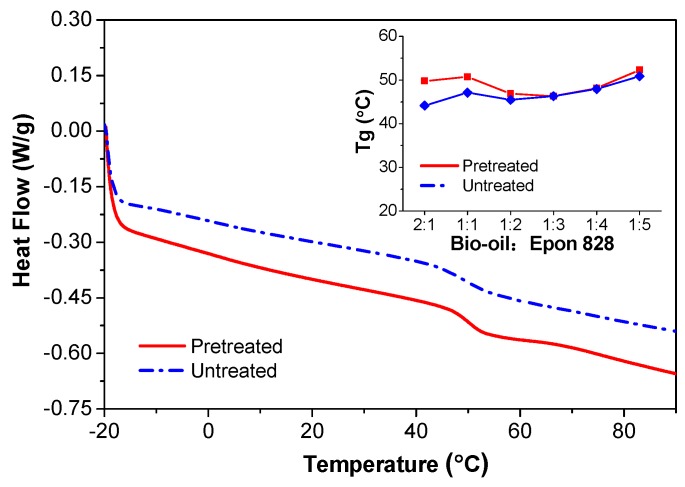
Glass transition temperature (*T*_g_) of cured bio-oil/epoxy resins at different synthetic ratios (inserted) and DSC curves of untreated and pretreated bio-resins at a 1:1 synthetic ratio.

The effect of acetone pretreatment on the glass transition temperature (*T*_g_) of the bio-oil/epoxy resins is demonstrated in Figure 6. A higher *T*_g_ was apparent with higher substitution rates of acetone pretreated bio-oil/epoxy resins. This illustrated that pretreatment in acetone can provide a higher thermal stability for the modified bio-oil/epoxy resins. Wu et al. observed a similar phenomenon [26]. They mixed liquefied wood with epoxy resin at various weight ratios and found that increasing the blended amount of liquefied wood would shift the peak of the curing reaction to a higher temperature but with less heat released. An assessment of the exothermic peaks within the polymer revealed similar cure behavior for the 1:1 ratio. All other ratios exhibited similar cure behavior with little change in cure rates or temperature. The higher *T*_g_ for the pretreated bio-oil/epoxy resins is perhaps also indicative of better cross-linking density as evidenced by the –OH and epoxied functional groups consumption (Figure 4).

### 3.5. TGA Measurement

**Figure 7:**
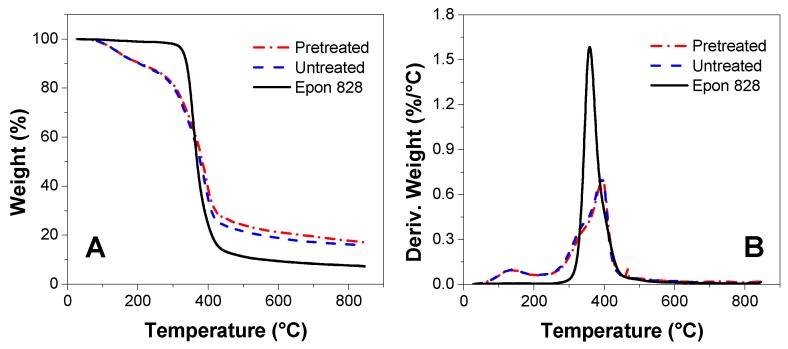
TGA (**A**) and derivative weight loss (DTG) (**B**) curves of Epon 828, and the untreated and pretreated bio-oil/epoxy resins at a 1:1 synthetic ratio.

The Epon 828, untreated and pretreated bio-oil/epoxy resins at a 1:1 ratio is demonstrated to compare the primary degradation temperature peaks (Figure 7). The TGA curve contained three stages for Epon 828, and the weight loss reached the maximum in the temperature range of 270 to 550 °C, which was around 90%. The TGA curve contained four stages for the bio-oil/epoxy resins. The first step was defined to occur between room temperature to 120 °C and this was attributable to the dehydration of water [46]. The weight loss during the dehydration stage was about 3% to 5%. Given that the water was removed during manufacture and in our lab, it is also possible that low organic weight materials volatilized at low temperatures. The weight loss nearly doubled to 9% in the second stage which spanned between 120 °C up to 230 °C. During the 3rd stage, the weight loss approached 65% at 500 °C and then a negligible loss of 5% occurred beyond 500 °C.

In the DTG figure, there was a maximum decomposition rate temperature at 358 °C for neat epoxy, which was attributable to the degradation of epoxy groups. The first temperature peak for the untreated and pretreated bio-oil/epoxy resins was essentially the same at 135 and 136 °C, respectively. This was attributable to the degradation of methylene bridges in the epoxy coupled with unreacted bio-oil components that decomposed into char residues and CO_2_, CO, CH_4_, CH_3_OH, and CH_3_COOH [47,48]. The overlapping peaks in the second degradation stage were also nearly the same at 393 and 394 °C for the untreated and pretreatment, respectively. This may be due to the bio-oil which contains macromolecule aromatic compounds, such as phenolic hydroxyls that reacted with the epoxy groups of Epon 828 resulting in better thermal stability of the modified resins. Additionally, since bio-oil possesses many compounds derived from lignin and polymerization may occur after pyrolysis, there may be an improvement in thermal stability since lignin requires a temperature greater than 380 °C for degradation [45].

According to Figure 7, the thermal stability of the pretreated 1:1 bio-oil/epoxy group did not change from that of the control group. Practically speaking, this would be beneficial during manufacturing where dialing in different levels of acetone would not visibly impact polymer thermal stability. Furthermore, it was earlier hypothesized that either (a) the acetone solvent would dissolve the bio-oil and would reduce the molecular group size resulting in improved cross-linking or (b) the molecules become more pliable with the addition of acetone resulting in an interpenetrating network. The improvement of polymer homogeneity with acetone pretreatment (Figure 3B) suggests a reduction in molecular group size was likely, but the possibility of an interpenetrating network has not been ruled out.

### 3.6. UV-Vis Spectra

**Figure 8:**
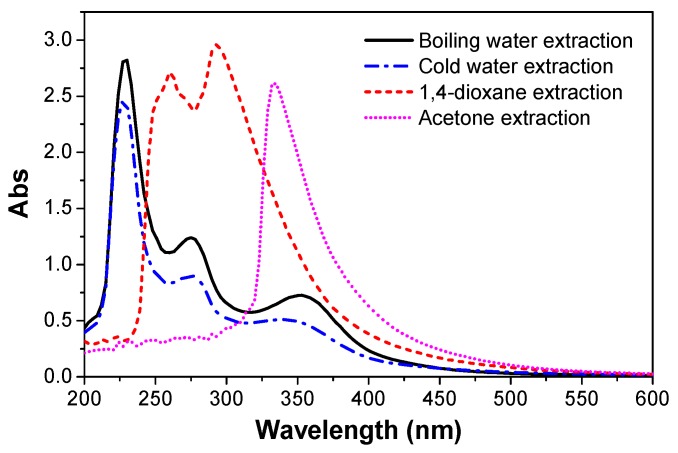
UV-Vis curves of extracts of pretreated bio-oil/epoxy resins.

The extracts of the pretreated bio-oil/epoxy resins after boiling water, cold water, acetone, and 1,4 dioxane extraction tests were collected and tested by UV-Vis (Figure 8). There was a strong absorption peak at 230 nm, a moderate absorption peak at 270 nm, and small absorption peak at 356 nm, which suggests the extracts contain an unsaturated aldehyde ketone conjugate system, and some aromatic rings. The strong absorption peaks at 260, 293, and 335 nm showed that multiple double bonds of the conjugate system (conjugated polyene) were contained in the extracts [49]. This means that some compounds within the bio-oil did not fully react with the epoxy system or the system was at least in an unsteady state. It appears that if we want to directly use wood pyrolysis bio-oil without any product separation for epoxy resin modification, it is better to remove the non-reactive fraction prior to blending and curing. On the other hand, the presence of extracts was far less than the bio-oil replacement rate (Figure 5). This indicates that some fraction of bio-oil containing phenols, aromatic hydrocarbons, and neutral components were substituted into the epoxy.

## 4. Conclusions

(1) The bond strengths and solvent resistance of the bio-oil/epoxy resins were improved after pretreatment for all bio-oil: epoxy ratios. However, as the replacement rate increased, so did the positive promotion effect of acetone on mechanical and thermal properties of the resin.
(2) The shear strength of the bio-oil/epoxy resins decreased as the bio-oil content increased. Increased bio-oil concentration resulted in decreased cross-linking density. However, after pretreatment of the bio-oil/epoxy mixture with acetone and then polymerization while curing, up to a 50% replacement was possible while still meeting usage requirements.
(3) The ATR-FTIR confirmed more –OH and –CH(O)CH– groups consumption in pretreated samples and a higher cross-linked structure after pretreatment of the bio-oil/epoxy mixture with acetone. DSC and TGA analysis demonstrated an improvement in thermal stability with acetone pretreatment. UV-Vis analysis revealed some potentially unreacted compounds within the bio-oil and this may have contributed to the weight loss observed when the cured polymer was exposed to various solvents.
(4) The pretreatment process coupled with precise tuning of the bio-oil to epoxy ratio was an effective method to control cross-linking and consequent bonding of the resin to the wood substrate.
(5) This study is industrially novel and relevant because the low variation in mechanical properties and polymer stability across a wide range of substitution rates will allow the manufacturer to adjust day-to-day changes in substitution rates without risk of a dramatic loss in material performance.
